# Automatic Nonnutritive Suck Waveform Discrimination and Feature Extraction in Preterm Infants

**DOI:** 10.1155/2019/7496591

**Published:** 2019-02-04

**Authors:** Chunxiao Liao, Austin O. Rosner, Jill L. Maron, Dongli Song, Steven M. Barlow

**Affiliations:** ^1^Department of Computer Science and Engineering, University of Nebraska-Lincoln, Lincoln, NE 68588-0115, USA; ^2^Mother Infant Research Institute, Tufts Medical Center, Boston, MA 02111, USA; ^3^Division of Neonatology, Department of Pediatrics, Santa Clara Valley Medical Center, San Jose, CA 95128, USA; ^4^Department of Communication Disorders, University of Nebraska-Lincoln, Lincoln, NE 68583-0738, USA; ^5^Department of Biological Systems Engineering, University of Nebraska-Lincoln, Lincoln, NE 68583-0726, USA; ^6^Center for Brain, Biology, and Behavior, University of Nebraska-Lincoln, Lincoln, NE 68588-0156, USA

## Abstract

*Background and Objective*: The emergence of the nonnutritive suck (NNS) pattern in preterm infants reflects the integrity of the brain and is used by clinicians in the neonatal intensive care unit (NICU) to assess feeding readiness and oromotor development. A critical need exists for an integrated software platform that provides NNS signal preprocessing, adaptive waveform discrimination, feature detection, and batch processing of big data sets across multiple NICU sites. Thus, the goal was to develop and describe a cross-platform graphical user interface (GUI) and terminal application known as NeoNNS for single and batch file time series and frequency-domain analyses of NNS compression pressure waveforms using analysis parameters derived from previous research on NNS dynamics. *Methods*. NeoNNS was implemented with Python and the Tkinter GUI package. The NNS signal-processing pipeline included a low-pass filter, asymmetric regression baseline correction, NNS peak detection, and NNS burst classification. Data visualizations and parametric analyses included time- and frequency-domain view, NNS spatiotemporal index view, and feature cluster analysis to model oral feeding readiness. *Results*. 568 suck assessment files sampled from 30 extremely preterm infants were processed in the batch mode (<50 minutes) to generate time- and frequency-domain analyses of infant NNS pressure waveform data. NNS cycle discrimination and NNS burst classification yield quantification of NNS waveform features as a function of postmenstrual age. Hierarchical cluster analysis (based on the Tsfresh python package and NeoNNS) revealed the capability to label NNS records for feeding readiness. *Conclusions*. NeoNNS provides a versatile software platform to rapidly quantify the dynamics of NNS development in time and frequency domains at cribside over repeated sessions for an individual baby or among large numbers of preterm infants at multiple hospital sites to support big data analytics. The hierarchical cluster feature analysis facilitates modeling of feeding readiness based on quantitative features of the NNS compression pressure waveform.

## 1. Introduction

Human neonates demonstrate two distinct types of sucking in a developmental progression: the first is nonnutritive sucking (NNS)—a repetitive bursting pattern characterized by mouthing and the tongue/jaw compressions on a pacifier or nipple in the absence of a liquid stimulus [1], and followed by nutritive sucking (NS)—when a nutrient is obtained from the bottle or breast. The NNS compression pressure pattern is an accessible motor behavior which can be digitized in real time and subsequently used by the medical care team to make inferences about brain development and prefeeding skills in preterm and term infants [2]. NNS is observable *in utero* as early as 12–18 weeks gestational age (GA) [3] with frequency-modulated bursts consisting of 2–13 suck cycles, separated by pause periods of 2–5 seconds to accommodate respiration [1, 4–7]. The modal frequency of NNS cycles is approximately 2 Hz [4]. Brainstem circuits involved in orofacial rhythmogenesis are modulated by sensory inputs, including cutaneous [8–17], olfactory [18–20], and auditory [21]. Initially, the NNS is not dependent on the respiratory phase, but an infant's continued experience with NNS facilitates the timing of swallows at “safe” points in the respiratory cycle which may be beneficial for nutritive feeding and safe swallows (e.g., end of inspiration or expiration) [22].

In the neonatal intensive care unit (NICU), the temporal organization of the NNS burst structure provides clinicians with diagnostic information on the infant's health status, including various forms of lung disease, infection, and neurological function during a critical period of brain development as the infant transitions from the tube to oral feeding. Prematurity itself can significantly alter developmental processes, as interruption of these critical periods of brain development can “impair fragile syntheses of central neural representations” of sensory and motor systems [23].

Physiological recordings of the nonnutritive and nutritive sucking pressure signals are becoming more common in the NICU with the advent of recording devices (analog and/or digital) to monitor and characterize basic patterning of ororhythmic activity associated with sucking and feeding in preterm infants [24–28]. Over the past 50 years, the literature is abound with descriptions of the general features of sucking behavior in preterm and term infants [1, 26, 29]. Recent innovations in feeding devices (modified bottles and/or nipples) also provide insights into suck patterning. For example, the Neonur nutritive sucking device is an advanced mobile nutritive sucking device that employs a feeding bottle unit with data acquisition of suck data acquired at 200 Hz and downloaded for offline processing using MATLAB software [25]. Analysis includes select temporal features, including the total number of sucks per 5-minute session, sucking duration, number of nutritive suck bursts, mean burst duration, within-burst suck frequency, and mean sucking pressure. Pressure amplitude pressure threshold criteria were used to discriminate suck and nonsucking movements. Another approach involved sampling intraoral pressure (suction) and expression (force associated with compression of the feeding nipple) during nutritive sucking [26, 27]. A small diameter polyethylene catheter with a closed-system silicone tube pressure sensor surface was positioned on the palatal surface of the nipple along with a second polyethylene fluted catheter tip placed proximal to the tip of the feeding nipple to sample “suction” during feeding by a neonate. The Mizuno laboratory has recorded intraoral pressure during nutritive sucking associated with the breast or bottle feeds using a commercially available 16-bit data acquisition module and bridge amplifier (ADInstruments, Inc., Colorado Springs, Colorado, USA) to condition the output signal from a disposable pressure transducer (Nihon Kohden, Tokyo, Japan). Simple descriptive measures of the suck waveform included hold and peak intraoral pressures, counts of suck cycles per burst, and duration of suck burst events [28].

Improvements in device designs and increased accessibility and testing of feeding readiness and feeding performance in newborns in the NICU translate to a proliferation of suck data to be analyzed for clinical and/or research purposes [26, 30, 31]. Thus, a need exists for an efficient software processing and analysis platform for automated extraction of salient NNS features in the time and frequency domains across treatment sessions, including advanced data analytics to support randomized multicenter clinical trials involving large numbers of preterm infants and repeated-measures acquisition of ororhythmic activity [32].

## 2. Materials and Methods

### 2.1. Software Design

We have developed NeoNNS to provide clinicians and developmental scientists with a powerful software platform tool for automatic NNS waveform discrimination and feature extraction in preterm infants during their hospitalization in the NICU. NeoNNS has been implemented Object Oriented Programming using Python language and the Tkinter package and supports Microsoft Windows 10 and Microsoft Access database.

### 2.2. Input and Output Data

For NeoNNS, source NNS assessment data files described in the present report were sampled from extremely preterm infants at three neonatal intensive care units (Boston, MA; Lincoln, NE; San Jose, CA) using the NTrainer System® (Innara Health, Inc., Olathe, Kansas USA), which is an FDA-approved medical device to promote NNS and facilitate the transition to oral feeds in preterm infants in the NICU. As new NNS source file formats are defined (i.e., binary, ASCII, etc.) and become available for other NNS recording systems, it will certainly be possible to import these suck waveform files to NeoNNS for NNS signal analysis.

The NTrainer System is currently used by more than 30 NICUs in the United States with one or two NTrainers operating at each NICU. Adoption of the NTrainer is expected to double in 2019 to include new NICUs in the US and international settings. The NTrainer has been and is currently used in randomized controlled trials (RCTs) to study the neurobiology of feeding [9, 10, 30, 33].

Neonatal practitioners record NNS assessment files to document a neonate's developmental progression for sucking in relation to the attainment of independent oral feeds. These NNS waveforms are digitized at 3 kHz (16-bits ADC resolution). Both the binary NNS assessment files (.assess) from the NTrainer System and a Microsoft Access database are needed to start the program. The length of each NNS assessment file is typically 540,000 samples (3 mins), although longer NNS assessment files can be processed easily. The GUI contains two modes, including single and batch file processing. For the single run mode, the intermediate files are redirected to a “single_intermediate” directory by selecting the desired signal processing operations, which in turn generates human readable text files that are converted from binary assessment files to peaks coordinate, bursts coordinates, power spectrum data, and features results. For the batch processing mode, the “intermediate” directory stores all the human readable text assessment files and the “result” directory is used to maintain other intermediate files for further analysis.

### 2.3. The Graphic User Interface

**Figure 1:**
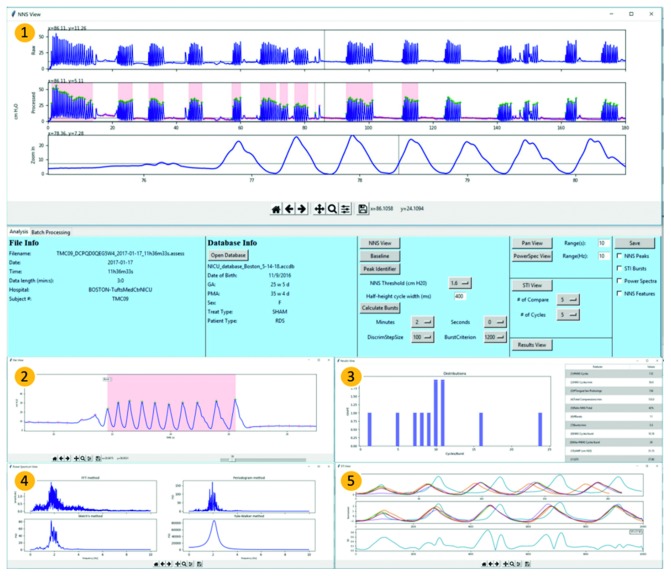
The graphical user interface of NeoNNS includes five pages: (1) NNS view; (2) Pan view; (3) Results view; (4) Power Spectrum view; (5) STI view.

NeoNNS can complete all phases of data preprocessing, suck pressure analysis, view intermediate results, and file saving. As shown in Figure 1, NeoNNS is implemented as five independent pages, including (1) NNS view, (2) Pan view, (3) Results view, (4) Power Spectrum view, and (5) STI view. Page descriptions are included in the following sections.

### 2.4. NNS View

After NNS assessment input file selection, the “NNS view” button is triggered to display the raw suck pressure signal in the top panel. The pressure baseline can been seen to vary over time due to the thermal drift induced by the infant's warm mouth on the instrumented pacifier which is connected to a closed-volume pneumatic sensing system. Clicking the “baseline correction” button generates a baseline-corrected plot in the middle panel. Next, the “peak identifier” function is used to discriminate NNS peaks from non-NNS peaks (i.e., chewing and tongue thrusts). The red index markers indicate non-NNS peaks, and the green index markers indicate NNS peaks detected by NeoNNS. In this step, two criteria need to be either selected or modified by the user to identify NNS pressure peaks. One is “NNS threshold,” which defines valid NNS pressure peaks with amplitudes greater than the default value of 1.6 cm·H_2_O (default threshold). The other is “half-height cycle width (ms),” which is the cycle width of pressure cycles at half height less than the default value of 400 milliseconds. Four parameter settings are included in NNS burst calculations, including minutes, seconds, DiscrimStepSize, and BurstCriterion. The combination of minutes and seconds define the length of the most active period of NNS production by the infant. DiscrimStepSize defines the size of a sliding window when searching the most active period from all the data samples. An NNS burst is defined as two or more NNS peak pressure events occurring within BurstCriterion distance with a default value equal to 1200 milliseconds. NNS bursts are highlighted as pink-colored blocks on the processed data plot panel. Detailed waveform characteristics using the zoom function in the third panel are realized by dragging the mouse over the desired pressure waveform segment on the second panel. Each panel has its own cursor with *x*- and *y*-coordinate indexes on the NNS waveform and can be referenced to canvas or data space. The icons on the tool bar contain picture editing and save functions, which can be used to format and create customized publication-quality graphics.

### 2.5. Pan View, Results View, and Power Spectrum View

The Pan view page (Labeled 2 in Figure 1) provides continuous full-screen zoom for users to find waveform targets of interest. The *X*-axis is scalable based on the range parameter and features a scroll bar to adjust waveform view. The bursts and indexes of each burst are automatically labeled. The Results view page (Labeled 3 in Figure 1) includes a summary of NNS feature results and an NNS burst cycle histogram for a given neonate at a specific PMA (days). NNS waveforms can be studied in the frequency domain on the Power Spectrum view page (Labeled 4 in Figure 1), which shows the results of 4 computational spectral methods, including the fast Fourier transformation (FFT) [34], periodogram [35], Welch's [36], and Yule–Walker methods [37], respectively. In the periodogram method, the significance of any possible periodic signals' frequencies has been calculated with a flattop window. In Welch's method, the power spectral density estimate is computed by dividing the best 2 minutes of the NNS signal into 90% of overlapped segments and applying a 50% length flattop window to prevent the leakage effect. The estimate of power spectrum density is calculated by averaging all the periodograms from each modified periodogram. The Yule–Walker method estimates the power spectral density by fitting the autoregressive model to the windowed (nominally at 50% of overall length) time-series data with the estimation order of 8. A high pass filter (*f*_c_ = 0.4 Hz) is applied before spectrum calculation to remove the DC offset.

### 2.6. STI View

Three panels are used to present the NNS spatiotemporal index (STI) visualization (Labeled 5 in Figure 1). After the first *N* bursts of *M* successive cycles are chosen, individual *N* bursts are aligned at the same origin as shown in the upper panel of STI view. The middle panel shows an overlay of five NNS bursts (*x*- and *y*-axis normalized) assigned to a 10,000 data sample window. The bottom plot panel shows the standard deviation of the *N* normalized burst segments from the second panel and displays the resulting STI value. All the intermediate results, including NNS peaks, STI, power spectrum, and related features, are saved in the .csv format.

### 2.7. Computational Methods

This section briefly describes the computational methods and analysis parameters implemented in NeoNNS. The algorithms, notations, and parameter specifications for time- and frequency-domain analysis routines described herein are based on previous research in preterm suck development [1, 4, 5, 8, 10, 15, 30, 32, 36, 37].

Default program settings defining the boundaries of NNS cycle geometry, NNS spatiotemporal index calculations, and Fourier transform of the NNS compression waveform were informed by these research works in the premature infant. A summary of parameters and their description is given in Table 1. Details of different parameters usage are as follows.

### 2.8. Calibration and Filter

NeoNNS automatically converts NNS assessment data files from voltage to cm·H_2_O-based on a 2-point calibration algorithm developed in our laboratory. The suck pressure signal is low-pass filtered (4-pole, digital Butterworth LP @ 50 Hz) to remove transients and high-frequency noise. NNS pressure waveform data are subsequently downsampled to 100 samples/second to improve memory resource management and computational throughput while preserving the fidelity of NNS waveform features for discrimination consistency.

### 2.9. Baseline Correction Pipeline

As described, NNS pressure signals are susceptible to thermal drift because of the infant's oral heat transfer on the silicone pacifier, which if left uncorrected, could impact the accuracy of NNS burst discrimination. Baseline variation is an important issue in many signal processing applications and can be addressed using baseline estimation or correction methods. Our NeoNNS application benefits from an asymmetric least-squares smoothing correction algorithm (ALSS) [40] iterated 10 times to automatically correct the nipple pressure signal baseline. Generally, a linear or nonlinear increase is added to the original signal, which causes data baseline drifting from zero to positive values. The ALSS algorithm effectively pulls all the lower points of every nipple pressure waveform back to the zero baseline while maintaining the structure of the suck compression waveform shape.

### 2.10. Suck Compression Peak Identification Methods

An automatic peak picker was designed to index and sort true-NNS pressure peaks from non-NNS events according to these rules: pressure peaks must exceed a user-defined pressure threshold (e.g., 1.6 cm·H_2_O) and meet a specified half-height pulse width criterion. Discriminated NNS cycles are labeled at their peaks with a green cross, and the non-NNS cycles are labeled with a red cross. The most active period of the NNS output (e.g., 2 mins) for any given data file is selected, and NNS bursts are automatically extracted and indexed according to their time order. An NNS burst is defined as 2 or more suck cycles satisfying user-defined cycle periods (e.g., <1200 ms). Individual NNS bursts are labeled by a pink-colored block, and the resultant burst distribution is calculated according to the number of NNS cycles per burst.

### 2.11. Feature Discrimination of NNS Waveforms

Eleven features based on the same parameter as introduced in the previous computation methods section are used to characterize ororhythmic motor activity in preterm infants, including the NNS burst structure and suck cycle dynamics during the most active 2 minutes (user-defined) within a 3-minute NNS assessment data file sampled cribside in the neonatal intensive care unit. These features include the following: (1) the number of NNS cycles is a tally of the number of discriminated NNS cycles during the most active 2 minutes, (2) NNS cycles per minute, and (3) the number of non-NNS events (the tongue and jaw posturing on the pacifier nipple) within the same analysis window. Tongue and jaw posturings on the pacifier nipple produce apparent changes in pressure signal amplitude but are considerably slower (lower spectral content), variable in morphology often with compression holds (biting) with relatively long-waveform half-heights intervals, and thus are readily distinguishable from an NNS cycle event, (4) total nipple compression events per minute, (5) ratio of NNS events compared to the total compression events expressed as a percentage, (6) number of NNS bursts during the most active 2 minutes (user-defined), (7) NNS bursts per minute, (8) NNS cycles per burst, (9) Max NNS cycles per burst, (10) Mean NNS cycle amplitude (cm·H_2_O), and (11) NNS spatiotemporal index (NNS STI). The default STI calculation is based on automatic selection of 5 NNS bursts each with 5 or more NNS cycles (default setting can be user-modified).

### 2.12. NNS Spatiotemporal Index (NNS STI)

**Figure 2:**
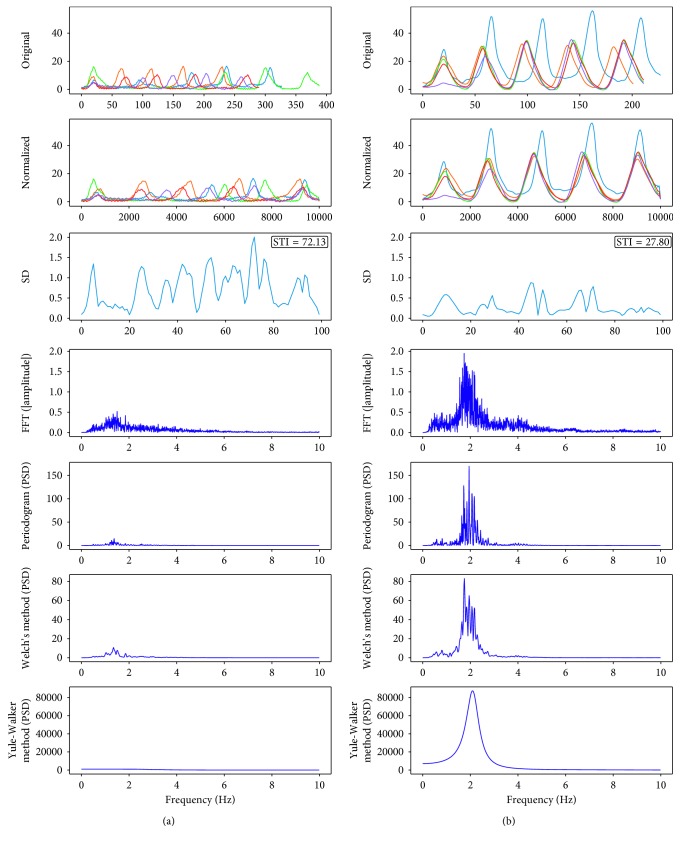
A comparison of NNS STI and spectral results for an extremely preterm infant (TMC09) at 231 days PMA (a) and 249 days PMA (b), respectively.

NNS STI is a quantitative measure of nonnutritive suck burst pattern formation [15]. The first *M* cycles from *N* successive bursts segments from the most active 2 minutes are interpolated into a 10,000 point length record and waveform amplitude is normalized to a *z*-score. The parameters *N* and *M* are defined by the user according to the NNS burst distribution profile. The sum of standard deviations is calculated at discrete 100 sample intervals [39] and can be plotted as a function of PMA days to visualize an infant's ororhythmic motor development in the NICU. For example, a relatively low STI value indicates good coregistration of suck cycle alignments during the burst production, whereas a higher STI value indicates poor coregistration of suck cycles and suggests the brainstem suck circuits are either underdeveloped or neurologic status is compromised. As shown for an extremely preterm infant (TMC09; Figure 2(a)), the coregistration of normalized NNS cycles among the 5 bursts is relatively poor with a resultant NNS STI of 72.13 at 231 days PMA. At 249 days PMA (Figure 2(b)), this same infant shows dramatic improvement in the NNS burst structure with an STI = 27.80. The spectral analyses shown in the bottom panels in Figure 2 reinforce this finding. The spectral peak for NNS activity sampled at 249 days PMA is significantly higher and 4 times larger in amplitude than NNS activity at 231 days PMA, and the entropy is lower. Thus, the combination of NNS STI and spectral analyses provide clinicians with lucid information on an infant's oromotor status in the NICU.

## 3. Results

### 3.1. Results Analysis of One Subject

**Figure 3:**
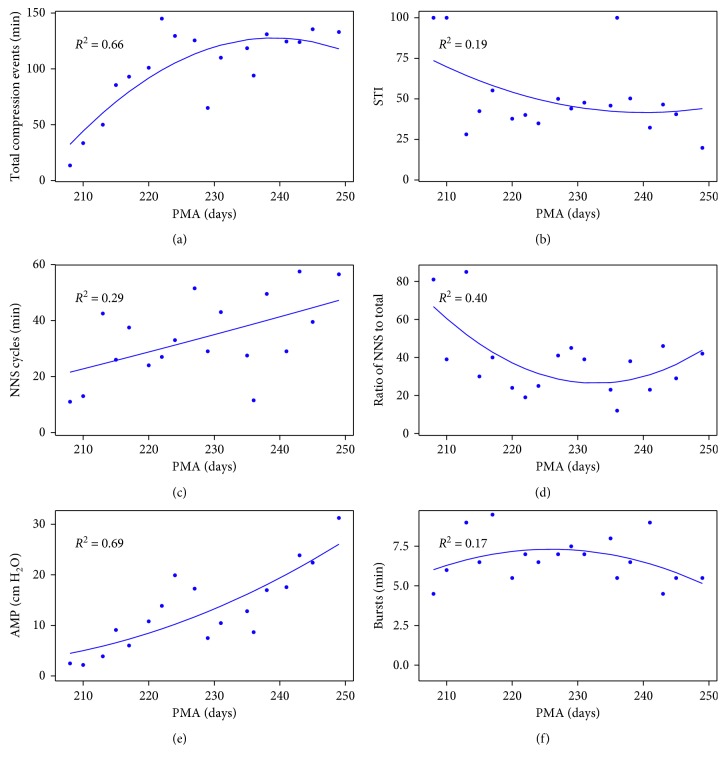
The correlation of six NNS features with PMA (days). Half-height cycle width = 500 ms; number of NNS bursts compare = 4; number of NNS cycles = 4.

As previously described, NeoNNS features two modes of operation: single file mode and the batch file-processing mode. The following example illustrates batch processing for a single infant over 18 repeated NNS assessment data files. The developmental trajectories for 6 NNS features which manifest significant trends as a function of PMA (days) are shown in Figure 3. Significant increases in the total number of compression cycles, NNS cycles/min, and NNS amplitude are evident, as well as a reduction in the NNS STI as this infant approached 249 days PMA.

### 3.2. Label Extraction

Label information, logged by the nursing staff into the NICU database, allows us to map the correspondence between feeding mode and associated NNS waveform files for any given infant. Preterm infants are fed by the tube or orally (bottle, breast) 8 times a day (3 hr feed cycles). The NICU database contains detailed information about feeding times, nutrient volumes, and feeding intake mode, which may involve oral (per os (this is from Latin, “per os,” means by the mouth) or PO (means by the mouth or orally)), nasogastric (NG), orogastric (OG), or combinations thereof. Individual NNS files were associated with time with the feeding information label. For example, if an infant's same day feeding type is PO, we assign the label “ready” for oral feeds to the corresponding NNS file (1 indicates “ready,” 0 indicates “not ready” for oral feeds, and 2 indicates “unknown”).

### 3.3. Association of Tsfresh Features with Clinical Data

Tsfresh is a Python package, which is used to automatically calculate a large number of time series characteristics or features. To evaluate the significance of Tsfresh features to characterize infant feeding readiness in the NICU, we conducted a comprehensive analysis assessing prediction performance and association with the clinical status at each repeated measurement of NNS activity. Tsfresh features were extracted from 568 NNS assessment files sampled from 30 preterm babies enrolled at 3 hospitals (Tufts Medical Center, Boston (MA), CHI St. Elizabeth's Health, Lincoln (NE), and Santa Clara Valley Medical Center, San Jose (CA)). The human subjects committees at each hospital approved the research protocol for this study. Written informed consent was obtained at each NICU prior to the participants' enrollment into the study.

**Figure 4:**
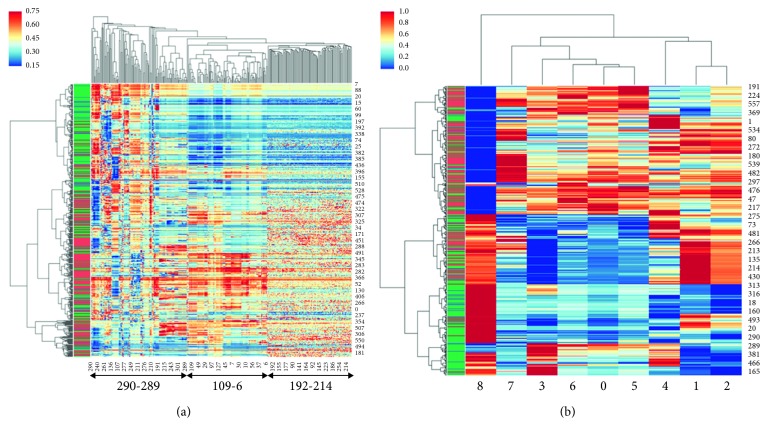
(a) Tsfresh cluster heat map of all 568 NNS files after feature elimination. *p* < 1.35*e* − 22. Red is positive, green is negative, and blue is unknown. The *x*-axis represents Tsfresh features, and the *y*-axis represents NNS assessment file records. The corresponding indexes mapping Tsfresh features (*x*-axis) and NNS files (*y*-axis) are saved in these complementary files: “mapping_heatmap_features.xlsx” and “mapping_heatmap_nns.xlsx.” (b) NeoNNS cluster heat map of all NNS files based on 11 NeoNNS features. *p* < 1.70*e* − 23. All the parameters are the same as used in the Tsfresh cluster heat map.

Hierarchical clustering illustrates the samples with similar NNS patterns (Figure 4) mapped according to the feeding mode. We calculated 789 Tsfresh features, which defined the characteristics for each NNS time-series data file. Readiness to feed was the major label for all the files. Here, we use the “readiness to feed” label to select Tsfresh features. The *p* value was used to quantify the prediction power of each Tsfresh feature, and the Benjamini and Yekutieli procedure is used to decide which Tsfresh features to keep [41]. After feature elimination, 310 Tsfresh features remained. The linkage between rows was computed with the Python Scipy.cluster.hierarychy library ward function [42], and a cluster heat map was generated with the Python Seaborn library clustermap function [43] with standard_scale = 0.

Labels were calculated by mapping the date between feeding and NNS records. After we obtain the labels, hierarchical clusters are built using the selected 310 Tsfresh features. Resultant classification accuracy is approximately 63%, and the false-positive ratio is 48%. The *χ* ^2^ test is performed based on *H*_0_, with clusters and readiness to feed as two independent variables. The significant association between assessment patterns with readiness to feed was demonstrated by a highly significant *χ* ^2^ test result, which was 95.68 and *p* < 1.35*e* − 22 for 2 clusters (ready to feed vs. not ready).

In the heat map shown in Figure 4(a), the *x*-axis represents Tsfresh feature indexes and *y*-axis represents NNS file indexes. The span marker between index 290 to 289 consists of 113 different statistical Tsfresh features, primarily peak and change quantiles information. The span marker between indexes 109 to 6 consists of an additional 96 Tsfresh features, which are mainly continuous wavelet transform coefficients and linear least-squares regression, and the last Tsfresh feature cluster is predominantly FFT coefficients. A comprehensive lookup table to map Tsfresh feature indexes to Tsfresh feature names and NNS file indexes to file names is available at https://github.com/cliao2/NNS-data.

### 3.4. Association of NeoNNS Features with Clinical Data

In our NeoNNS application, we generated 11 features which have medical significance to discriminate the feeding readiness among preterm infants. To demonstrate how our NeoNNS features performance in readiness to feed labels classification, all 9/11 NeoNNS features (because two features are dependent) are used to build the heat map with the same preprocessing parameters with the Tsfresh heat map generation process. As shown in Figure 4(b), the two feeding mode classes can be largely separated by NeoNNS features with classification accuracy greater than 67% and false-positive ratio is 28%. The highly significant *χ* ^2^ test result is 99.78 and *p* < 1.70*e* − 23 for 2 clusters. Both NeoNNS features and Tsfresh features demonstrate our prediction power using clinical data sampled from preterm infants. As shown in Figures 4(a) and 4(b), the comparison of the two-cluster analyses demonstrates that the NeoNNS application is robust in implementing data preprocessing and NeoNNS feature generation algorithms. Secondly, the 9/11 features from NeoNNS application achieved better classification accuracy than the 310 Tsfresh features. This comparison illustrates NeoNNS's effectiveness in discrimination and detection of oral feeding readiness based on our algorithms for analysis of NNS waveform features among a cohort of extremely premature infants.

### 3.5. Single NeoNNS Feature Distribution and Pairwise NeoNNS Features Distribution between Positive and Negative Data

**Figure 5:**
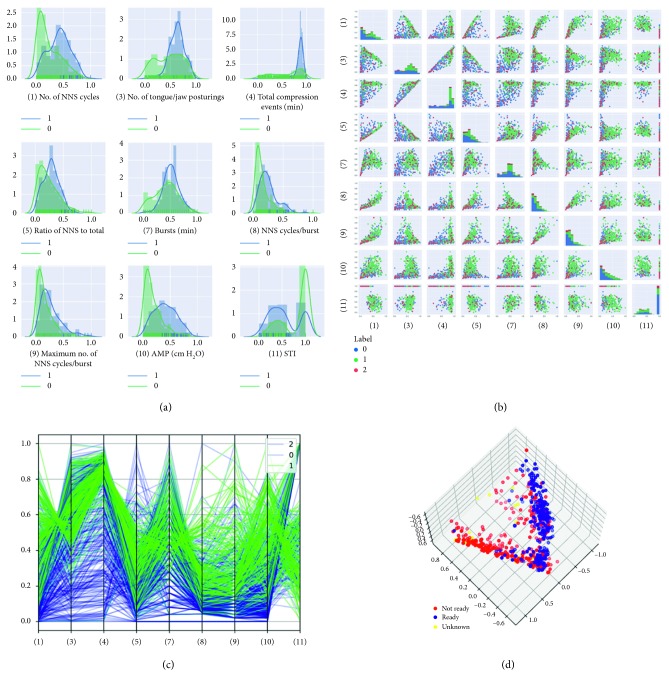
Results analysis. (a) NeoNNS features distribution between positive (labeled as “1” means ready for oral feed or “0” not ready for oral feed); (b) the pairwise distribution between every two NNS NeoNNS features; (c) a parallel coordinate feature view of our 9 NeoNNS features. The number inside parentheses represents the feature indexes. Green signifies positive (ready to feed) records, and blue is negative (not ready). (d) PCA plot of 3 components (infant feed modes), where red dots are negative (not ready to orally feed), blue signifies positive (ready to orally feed), and yellow is unknown oral feeding readiness.

To explore how each NeoNNS feature discriminates between two classes of feeding readiness (positive and negative), single feature density and counts distribution plots are included in Figure 5(a). Among 11 features, two features are dependent with other features, so only 9 out of 11 features are used to perform the experiment. All features have been normalized to a range from 0 to 1. The *y*-axis indicates counts of each feature value. Figure 5(a) also includes both counts distribution of each discrete feature value and continuous density distribution profile for all 568 NNS records. A GLM ANOVA, completed using Feature_value as the response variable, found highly significant main effects for the factors Feature_Type (*F*(8,4742) = 1491.50, *p* < 0.0001) and feeding mode (*F*(1,4742) = 251.94, *p* < 0.0001) (Tables 2 and 3). All 9 features have a significant effect on the prediction of readiness to feed. Figure 5(b) shows the pairwise correlation plots between every two features. Three colors are used to label different data: blue is “ready;” green is “not ready;” and red is “unknown.”

### 3.6. Parallel Coordinates Visualization

To visualize all the NeoNNS feature patterns in all dimensions without information loss, parallel coordinates was used to plot all the NeoNNS features. As shown in Figure 5(c), the positive and negative records generally have the same trend for each feature. However, the value of most of positive features is greater than the negative features. The positive features are well organized compared to the negative features. A likely reason is the blue color contains both known “not ready” and unknown records.

### 3.7. PCA Analysis

Principal component analysis (PCA) was performed for advanced exploration of the NNS data in relation to feeding readiness. It is apparent from Figure 5(d) that a hyperplane separates two point clouds, but not our class labels. All the positive points are in one cluster with some negative, and the other cluster is purely negative. This means our label mapping causes the false-negative issue, since the NICU nurses tend to only feed using the PO mode when the neonate is very ready. During other times, the neonate probably was ready for PO but was tube-fed instead, resulting in a false negative. This analysis reinforces the main purpose of our research, that is, to label the NNS data for feeding readiness.

## 4. Discussion and Conclusions

NeoNNS software is not instrument dependent; rather, it has the potential to analyze nonnutritive suck pressure signals from devices other than the NTrainer System as long as the source data files are digitally formatted as a time series of nipple pressure indexes with a specified sampling rate and calibration factor. In our current database, the feeding mode for any given infant is decided by NICU nurse's observation and prediction, which is subject to false-negative labelling. This limitation not only causes the NeoNNS software computational result difficult to justify but also decreases the accuracy of the batch data cluster classification. Future work with much larger data sets from our participating NICU network will focus on improving the labelling of NNS data for oral feeding readiness. When we can accurately label all data, it will be possible to create a prediction model of oral feeding readiness from virtually any unknown NNS record. The default analysis parameters implemented for this software are derived from peer-reviewed publications on NNS dynamics in preterm infants. As a research tool, NeoNNS provides clinical investigators with the added flexibility of modifying the default settings to explore new hypotheses and questions as they relate to differences in NNS burst structure as a function of the disease state, postmenstrual age, and experimental interventions.

This NeoNNS software application makes big data analysis possible and efficient for NICU practitioners and scientists. Our Python-based NNS waveform discrimination and feature extraction software offers rapid and comprehensive measurements in the time and frequency domains and modeling of NNS pressure dynamics in preterm infants as a function of PMA across repeated sessions, among large cohorts, and across multiple neonatal intensive care units. Our experiments from Tsfresh and NeoNNS features cluster processing both illustrate the significant prediction capability for oral feeding readiness that is possible from our data analytics pipeline using a neonate's NNS compression pressure waveform as the input.

## Figures and Tables

**Table 1:** Summary of parameters used by the NeoNNS application.

	Units	Description	Reference
NNS threshold	(cm·H_2_O)	NNS peaks with amplitude greater than this value used as feature extraction	[10]
Half-height cycle width	(ms)	NNS cycle width at half-height less than this criterion used as feature extraction	[38]
Minutes/seconds	(min/s)	The time epoch of the most active NNS period	[10]
DiscrimStepSize	(samples)	Sliding window size to localize the most active period of sucking	
BurstCriterion	(ms)	Two or more NNS events occurring within this value	[10]
Number of compare		Number of NNS bursts	[39]
Number of cycles		Number of successive NNS cycles	[39]

**Table 2:** GLM analysis of variance.

Source	DF	Adj. SS	Adj. MS	*F* value	*p* value
Feature_type	8	12192433	1524054	1491.50	0.000
OralFeed	1	257443	257443	251.94	0.000
Error	4742	4845517	1022		
Lack of fit	8	506013	63252	69.00	0.000
Pure error	4734	4339504	917		
Total	4751	17295392			

**Table 3:** GLM fitted means.

Term	Fitted mean	SE mean
*Feature_type*		
1	56.74	1.39
3	161.64	1.39
4	109.19	1.39
5	27.02	1.39
7	6.80	1.39
8	5.65	1.39
9	11.57	1.39
10	24.75	1.39
11	76.55	1.39
*OralFeed*		
0	45.890	0.614
1	60.758	0.707

## Data Availability

The binary nonnutritive suck waveform data used to support the findings of this study are restricted by the University of Nebraska Human Subjects Committee in order to protect patient privacy as this software application is part of an ongoing randomized clinical trial sponsored by the National Institutes of Health (R01 HD086088). Data from this NIH trial are registered at ClinicalTrials.gov (RCT # NCT02696343). A sample of deidentified binary nonnutritive suck waveform files has been included as supplementary information files.

## Supplementary Materials

Supplementary Materials Three deidentified binary nonnutritive suck waveform files have been included as supplementary materials. They are from one subject with PMA of 34 weeks 5 days, 35 weeks, and 35 weeks 4 days, respectively.
